# Hepatocyte Produced Matrix Metalloproteinases Are Regulated by CD147 in Liver Fibrogenesis

**DOI:** 10.1371/journal.pone.0090571

**Published:** 2014-07-30

**Authors:** Sarah R. Calabro, Annette E. Maczurek, Alison J. Morgan, Thomas Tu, Victoria W. Wen, Christine Yee, Auvro Mridha, Maggie Lee, William d'Avigdor, Stephen A. Locarnini, Geoffrey W. McCaughan, Fiona J. Warner, Susan V. McLennan, Nicholas A. Shackel

**Affiliations:** 1 Liver Cell Biology, Centenary Institute, Sydney, NSW, Australia; 2 Sydney Medical School, The University of Sydney, Sydney, NSW, Australia; 3 Victorian Infectious Disease Laboratory, Melbourne, VIC, Australia; 4 Department of Endocrinology, Royal Prince Alfred Hospital, Camperdown, Sydney, NSW, Australia; 5 A.W. Morrow Gastroenterology and Liver Centre, Royal Prince Alfred Hospital, Camperdown, Sydney, NSW, Australia; 6 Liver Injury and Cancer, Centenary Institute, Sydney, NSW, Australia; University of Sydney, Australia

## Abstract

**Background:**

The classical paradigm of liver injury asserts that hepatic stellate cells (HSC) produce, remodel and turnover the abnormal extracellular matrix (ECM) of fibrosis via matrix metalloproteinases (MMPs). In extrahepatic tissues MMP production is regulated by a number of mechanisms including expression of the glycoprotein CD147. Previously, we have shown that CD147 is expressed on hepatocytes but not within the fibrotic septa in cirrhosis [1]. Therefore, we investigated if hepatocytes produce MMPs, regulated by CD147, which are capable of remodelling fibrotic ECM independent of the HSC.

**Methods:**

Non-diseased, fibrotic and cirrhotic livers were examined for MMP activity and markers of fibrosis in humans and mice. CD147 expression and MMP activity were co-localised by *in-situ* zymography. The role of CD147 was studied *in-vitro* with siRNA to CD147 in hepatocytes and *in-vivo* in mice with CCl_4_ induced liver injury using **ã**CD147 antibody intervention.

**Results:**

In liver fibrosis in both human and mouse tissue MMP expression and activity (MMP-2, -9, -13 and -14) increased with progressive injury and localised to hepatocytes. Additionally, as expected, MMPs were abundantly expressed by activated HSC. Further, with progressive fibrosis there was expression of CD147, which localised to hepatocytes but not to HSC. Functionally significant *in-vitro* regulation of hepatocyte MMP production by CD147 was demonstrated using siRNA to CD147 that decreased hepatocyte MMP-2 and -9 expression/activity. Further, *in-vivo* α-CD147 antibody intervention decreased liver MMP-2, -9, -13, -14, TGF-β and α-SMA expression in CCl_4_ treated mice compared to controls.

**Conclusion:**

We have shown that hepatocytes produce active MMPs and that the glycoprotein CD147 regulates hepatocyte MMP expression. Targeting CD147 regulates hepatocyte MMP production both *in-vitro* and *in-vivo*, with the net result being reduced fibrotic matrix turnover *in-vivo*. Therefore, CD147 regulation of hepatocyte MMP is a novel pathway that could be targeted by future anti-fibrogenic agents.

## Introduction

Regardless of the aetiology of chronic liver injury, a canonical pathway of fibrosis development results in progressively abnormal matrix deposition and eventual cirrhosis with the sequelae including liver failure and hepatocellular carcinoma (HCC) [2], [3]. Chronic fibrotic liver injury is an active process characterised by abnormal extracellular matrix (ECM) deposition and remodelling [2], [3]. Matrix metalloproteinases (MMPs) are proteolytic enzymes, which play an important role in all stages of progressive liver injury from fibrogenesis initiation through to resolution [4]–[9]. MMPs are abundantly produced by hepatic stellate cells (HSC) within the dense fibrotic bands, which surround nodules of hepatocytes. Further, MMPs are also secreted by other intrahepatic cell populations including inflammatory cells and hepatocytes [10]–[12]. However, the role of MMPs originating from non-HSC intrahepatic cell populations, such as hepatocytes [12], has not until recently been attributed a significant role in the ECM remodelling associated with progressive fibrosis. MMP-10 has been shown to be expressed by hepatocytes, cholangiocytes and macrophages and can clearly alter fibrogenesis in a non-HSC dependent manner [13]. However, functional studies of other MMPs in non-HSC cell populations are lacking. In particular, studies of the role of the hepatocyte in intrahepatic fibrogenesis are comparatively sparse and the hepatocyte, the main parenchymal cell of the liver, is not considered to have a functionally significant role in either ECM production or remodelling. Indeed it is widely asserted that hepatocytes are “innocent bystanders” which release products of cell apoptosis, necrosis, or secrete chemokines to attract inflammatory cells and activate HSC that are responsible for the production of the abnormal matrix, MMPs and the remodelling of the ECM [2]. It is now apparent that the HSC makes the abnormal matrix with progressive fibrosis but other intrahepatic cell populations are, in addition to the HSC, capable of remodelling the ECM [2], [3], [13].

We have reported that CD147, also known as Extracellular Matrix Metalloproteinase Inducer (EMMPRIN) or basigin (Bsg), is increased in cirrhotic liver and localised to the membrane of hepatocytes [1], [14]. Importantly, CD147 is not expressed within the fibrotic septa where there is a predominance of activated HSC [1], [14]. CD147 is a widely-expressed multifunctional, highly glycosylated, cell surface transmembrane protein which is upregulated in many forms of tissue injury associated with inflammation and matrix remodelling [15]–[17]. In multiple extrahepatic organ systems and in inflammatory conditions such as arthritis, CD147 has been shown to regulate MMP production and determine the progression of fibrosis [14], [18]–[24]. The functional role of intrahepatic CD147 has been studied and it has been variably co-localised with the HSC-marker **α**-SMA and therefore has been implicated in HSC activation [24]. However, this study used antibody HAb18G which is not available commercially and our studies with established commercial antibodies, including those available from widely adopted hybridomas [25], show that CD147 is abundantly expressed on hepatocytes but not HSC [14]. The discrepancies in these studies are likely due to both isoforms and glycoforms of the protein that have differing biological activities [14], [24], [26]. Therefore, based on previous studies showing hepatocyte MMP production [12], [13] combined with our demonstrated changes in hepatocyte expression of CD147 with liver injury [1], [14] we hypothesise that: *In response to injury hepatocytes produce MMPs regulated by CD147 and thereby directly contribute to intrahepatic ECM remodelling, independent of the HSC*. The data presented in this manuscript supports this hypothesis and demonstrates that the hepatocyte production of active MMPs is regulated by CD147. Importantly, this is clearly functionally significant as *in-vivo* α-CD147 interventions alter fibrotic liver injury.

## Experimental Procedures

### Ethics Statement

Human tissues samples were obtained from Royal Prince Alfred Hospital, Sydney with approval of Human Research Ethics Committee (X10-0072). Human tissue used in this study was previously utilized for research [1], [27]. Informed written consent was obtained from all participants. The ethics committee waived the need for written consent for use of donor tissue. In Australia, the ethics of human research is governed by the National Statement on Ethical Conduct in Human Research (2007) issued by the National Health and Medical Research Council (NHMRC). Under these guidelines all research involving humans requires ethical approval.

Animal experiments were performed in accordance with Sydney University Animal Ethics Committee requirements (K75/10-2008/3/4801). The Australian Code of Practice for the Care and Use of Animals for Scientific Purposes was followed. This includes a responsibility to protect and promote the welfare of animals used.

We confirm that Sydney University Animal Ethics Committee specifically approved the animal part of our study. The Code of Practice embodies the principles of: Reduction of animal use, Replacement of animal use and Refinement of animal use. These are known as the "3 Rs". It is important to consider these principles when designing and carrying out projects.

### Human Tissue and Cell Lines

Non-diseased donor and end-stage cirrhotic liver tissues were collected from patients attending Prince Alfred Hospital, Sydney during liver transplantation. pH5CH8 cells were kindly provided by Prof. Li [28], [29].

### Mouse Studies and Primary Hepatocyte Isolation

*Balb*/c and *C57bl/6* mice were used for *in-vivo* studies [30], [31]. We have elected to use the two mouse backgrounds, as they are known to have differing fibrotic responses [32]. Liver injury was induced with carbon tetrachloride (CCl_4_). For the CCl_4_ model mice were injected twice weekly for upto four weeks with 100 µl of 12% v/v CCl_4_ in paraffin oil i.p, control mice only received paraffin oil (Ajax Finechem). The role of CD147 was examined using an α-CD147 blocking antibody (mAb clone RL73.2) produced and purified as previously described [33]. The antibody was administered (i.p 100 µg) twice weekly. Mice treated with CCl_4_ and administered IgG2a (100 µg, HB-189, ATCC) were used as controls. At termination animals were euthanized and blood was obtained by cardiac puncture and used for measurement of aspartate transaminase (AST). Livers were collected for histological studies, measurement of MMP activity and expression of genes of interest by quantitative PCR. Hepatocytes were isolated using a two-step collagenase perfusion technique [34] and gene expression levels were measured by quantitative PCR. Viability was greater than 95% for isolated hepatocytes at 48 hours in culture. Contamination of the hepatocyte preparations with Kupffer cells/macrophages was assessed by F4/80 staining and morphology. Hepatocyte purity was consistently found to be greater than 95%.

### Histochemistry

Paraffin embedded human liver tissue from controls, non-diseased donor samples, or subjects with end-stage cirrhosis caused by primary biliary cirrhosis (PBC), primary sclerosing cholangitis (PSC), alcoholic liver disease (ALD), autoimmune hepatitis (AIH) and hepatitis C (HCV) taken at time of liver transplantation were examined. The following antibodies were used: α-CD147 antibody (clone MEM-M6/1, Abcam), α-MMP-1 (clone 41-1E5, Calbiochem), α-MMP-2 and α-MMP-9 (clone 8B4 and polyclonal antibody H-129 Santa Cruz) or mouse IgG1 (MOPC 21, Abcam) isotype control. Goat α-mouse-HRP and goat α-rabbit-HRP (Dako) and NovaRED (Vector Labs) or DAB (Sigma) were used for detection. Sections were counterstained with Haematoxylin and Eosin (H&E).

Co-localisation studies were performed on 5 µm frozen and fixed (acetone:methanol 1∶1) sections using α-CD147-FITC (clone MEM-M6/1, Abcam) in combination with α-cytokeratin 18 (CK18, clone DC10), α-CK19 (clone RCK108), α-CD45 (clone 2B11+PD7/26) or α-CD31 (clone JC70A) all from Dako Cytomation and α-SMA-Cy3 (clone 1A4 from Sigma). AlexaFluor goat **α**-mouse 594 was used as secondary antibody (Lifetechnologies). All sections were imaged by confocal microscopy.

H&E and Pico-Sirius Red (PSR) staining were used to investigate tissue morphology and collagen content in paraffin-embedded sections. Sections were cut and stained by the University of Sydney Pathology Department.

### Pico-Sirius Red Quantification

Total liver sections stained with PSR were imaged using a Leica DM6000B with LAS Power Mosaic (Leica, Germany) at a magnification of 10×. Image J 1.48J was used for quantification of PSR staining. Briefly, five regions of interests (ROI, 1386 µm ×1316 µm) per section were randomly selected, avoiding large blood vessels and empty spaces. The images were converted into Red-Green-Blue Stacks and the thresholds of the green channel were equally adjusted for all images. The positive area fraction for all ROIs was measured using Image J. Sections of at least four animals (n = 4−8) were analysed for each treatment condition.

### Determination and Localisation of MMP Activity

The cellular localisation of MMP activity was examined on frozen, unfixed liver sections using the following liver cell markers CK18, α-SMA-Cy3 or CD147 as described above. AlexaFluor goat α-mouse 594 was used to visualise CK18 and CD147, nuclei were visualized using DAPI. MMP activity in human liver tissue was subsequently studied by *in-situ* zymography. Antibody stained sections were overlayed with agarose (1%w/v) containing quenched-fluorescent (DQ) gelatin (1 mg/ml, Lifetechnologies). The sections were then incubated for 2.5 hrs at 37°C before imaging by confocal microscopy (Leica SP5). In a parallel series, sections were overlayed with agarose containing DQ gelatin and aminophenyl-mercuric acetate (7 µM, APMA) to activate all MMPs [35], [36]. Sections overlayed with agarose only, or DQ agarose containing the MMP inhibitor 1,10-phenanthroline (20 mM, Sigma) were studied as control. Gelatin zymography was used to measure the pro- and active forms of MMP-2 and MMP-9 in serum-free conditioned media using equal protein concentrations, as determined by DC protein assay (Biorad) [37]. Results are expressed relative compared to control.

### Knockdown of CD147 Expression using siRNA

The effect of knockdown CD147 protein expression was examined in the human hepatocyte cell line pH 5CH8. Cells were grown in DMEM and FCS (10%v/v) as previously described [28], [29]. At 70% confluence, 1×10^5^ cells were transfected with either 100 pmol scrambled siRNA (5′-GAAATCTGCCAACGCACTAAA-3′) or siRNA targeting CD147 (siCD147, 5′-AAGTCGTCAGAACACATCAAC-3′) using Lipofectamine 2000 (Lifetechnologies) according to the manufacturers' protocol. Cells in serum-free medium (0.1% BSA) were incubated for 48 hrs before MMP expression was induced by addition of 10 ng/ µl hTNF (Peprotech). Forty-eight hours later the conditioned media and cell pellets were collected to measure total MMP activity, and protein expression of MMP-2, MMP-9, MMP-14 and CD147.

### Western Blot

Cells were homogenised in NP-40 sample buffer. After incubation on ice for 20 mins, the lysates were centrifuged (3 mins at 15,000×*g*) and the protein concentration was determined using the DC protein assay (Biorad). Proteins were separated by electrophoresis on 4–20% Bis-Tris NuPAGE gels (Lifetechnologies) and transferred to PVDF membranes prior to immunoblotting with **α**-CD147 (clone ZMD.182, Zymed), **α**-MMP-14 (clone EP1264Y, Abcam, US) or **α**-GAPDH (clone ZG003, Lifetechnologies) antibodies. Blots were incubated with α-mouse-HRP or **α**-rabbit-HRP and visualised with Immobilon Western Chemiluminescent HRP Substrate (Millipore). Relative protein expression was determined by densitometry using Image J software program and normalised to GAPDH.

### Quantitative PCR

Total RNA from human and mouse livers or snap-frozen mouse hepatocytes was isolated with TRIzol and cDNA was synthesised using SuperScript III Reverse Transcriptase (all Lifetechnologies). Transcripts were quantified using either specific Taqman probes (Table S1, Lifetechnologies) according to the manufacturers' instructions or SensiMix SYBR Low-ROX Kit (Bioline). Primer sequences for SYBR assays are shown in Table S2. For SYBR qPCR the reaction was activated by incubation at 95°C for 10 mins followed by 40 cycles of 15 secs at 95°C and 60 secs at 60°C. Relative mRNA expression was determined by normalisation to **β**-Actin, 18 S and 36B4.

### Hydroxyproline Assay

Hydroxyproline content of mouse liver tissue was measured as a marker of net ECM deposition as previously described [38]. Briefly, duplicate samples of liver tissue (60 mg) were hydrolysed in 1.5 ml of 6 M HCl at 110°C overnight. Cooled samples were diluted to 6 ml in *d*H_2_O and adjusted to pH 7.4 before incubation with activated charcoal (Ajax Finechem). After 30 mins the samples were filtered (Whatman No. 3) and further diluted to 12 ml in dH_2_O. Two hundred μl sample were combined with 400 µl isopropanol and 200 µl chloramide T (308 mM) for 5 mins. Ehrlich's solution (2.5 ml) was added and the samples were incubated at 65°C for 25 mins before being cooled. Sample aliquots of 200 µl were then transferred to a 96-well plate and the absorbance was measured at 570 nm (POLARstar Omega; BMG Labtech). Hydroxyproline concentration was calculated using a hydroxyproline standard (Fluka Chemicals) and normalised for starting tissue weight.

### Data Analysis

Except where otherwise indicated statistical analysis was performed using Mann-Whitney U t-test. Significance was accepted at p<0.05. All data is presented as mean ± SEM and expressed as fold change over control.

## Results

We initially studied expression of MMP activity, protein and mRNA in fibrotic liver injury and then studied expression of the known MMP regulator CD147. Subsequently, we have described the functional CD147 regulation of MMP activity in fibrotic liver injury.

### Matrix Metalloproteinases in Human Liver Disease

**Figure 1:**
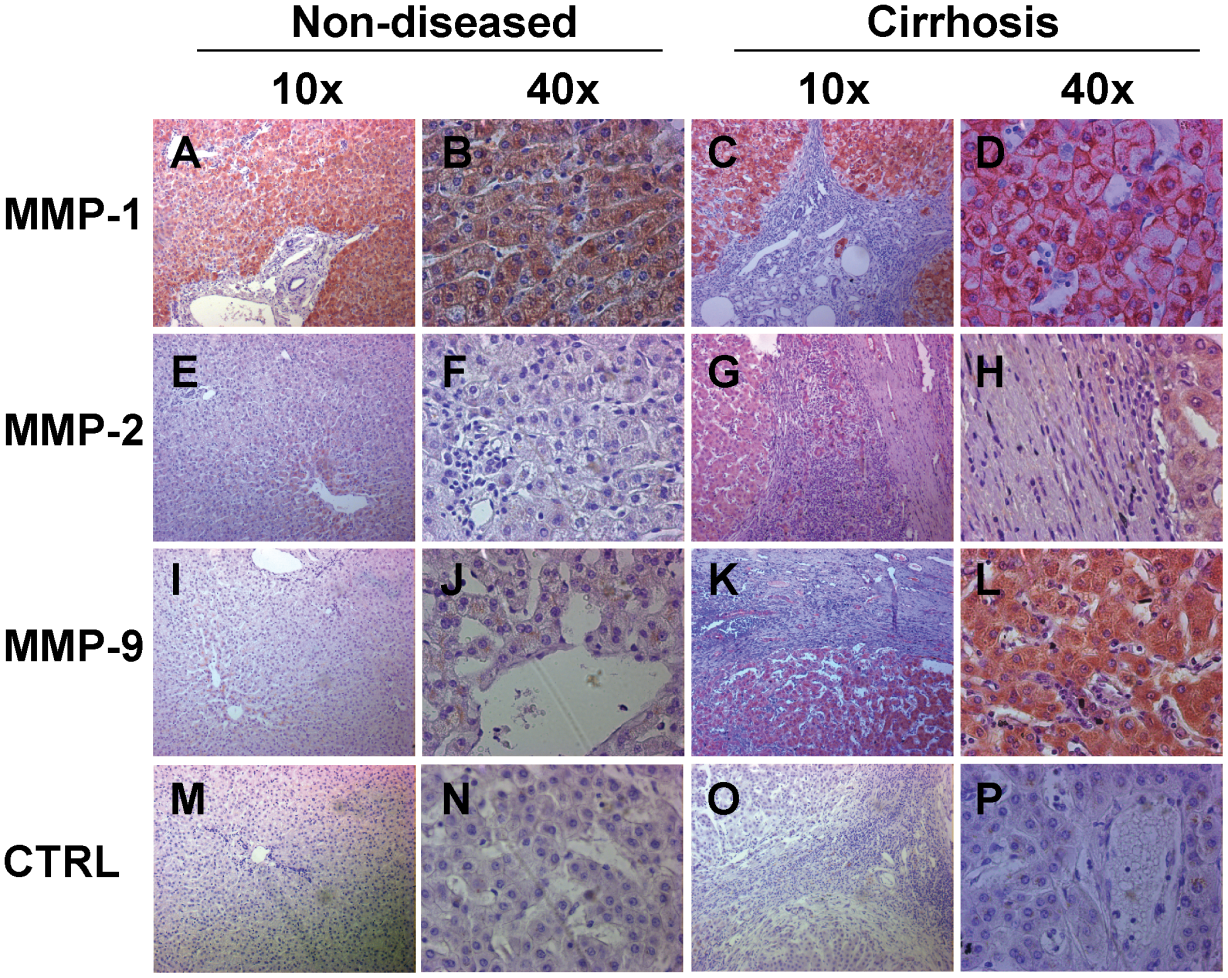
Immunohistochemistry of MMP-1, MMP-2 and MMP-9 expression in non-diseased and cirrhotic human liver injury. Immunohistochemistry with NovaRED detection is shown for both non-diseased donor (Panels A, B, E, F, I, J, M and N) and cirrhotic HCV explant tissue (Panels C, D, G, H, K, L, O and P). The same result was seen for PBC, PSC, AIH and ALD tissue (not shown). MMP-1 was found both in non-diseased and cirrhosis in both hepatocytes and biliary structures located in the fibrous septa (Panels A–D). MMP-2 expression was not significant in non-diseased tissue (Panels E and F) but was seen in hepatocytes, bile ducts and HSC (Panels G and H) of cirrhotic tissue. A similar pattern was also observed for MMP-9 expression (Panels I-L). The isotype controls showed no significant staining (Panels M-P). Magnification is 10× and 40×.

To investigate the expression of MMPs during liver injury we stained non-diseased and end-stage cirrhotic human liver sections with antibodies for MMP-1, MMP-2 and MMP-9 (Figure 1). We found expression of MMP-1 in non-diseased (Figure 1, Panels A, B) and cirrhotic tissue (Figure 1, Panels C, D), while MMP-2 and MMP-9 expression could only be detected in cirrhotic explant tissue (Figure 1, Panels G, H, K and L) but not in non-diseased tissue (Figure 1, Panels E, F, I and J). MMP expression was found in hepatocytes as well as in the fibrous septa. No appreciable staining was seen in the isotype antibody controls (Figure 1, Panels M-P). As IHC cannot distinguish if non-septal HSC are positive for CD147 or active MMPs, we proceeded to examine CD147 expression and MMP activity by confocal immunofluorescence in combination with *in-situ* zymography.

**Figure 2:**
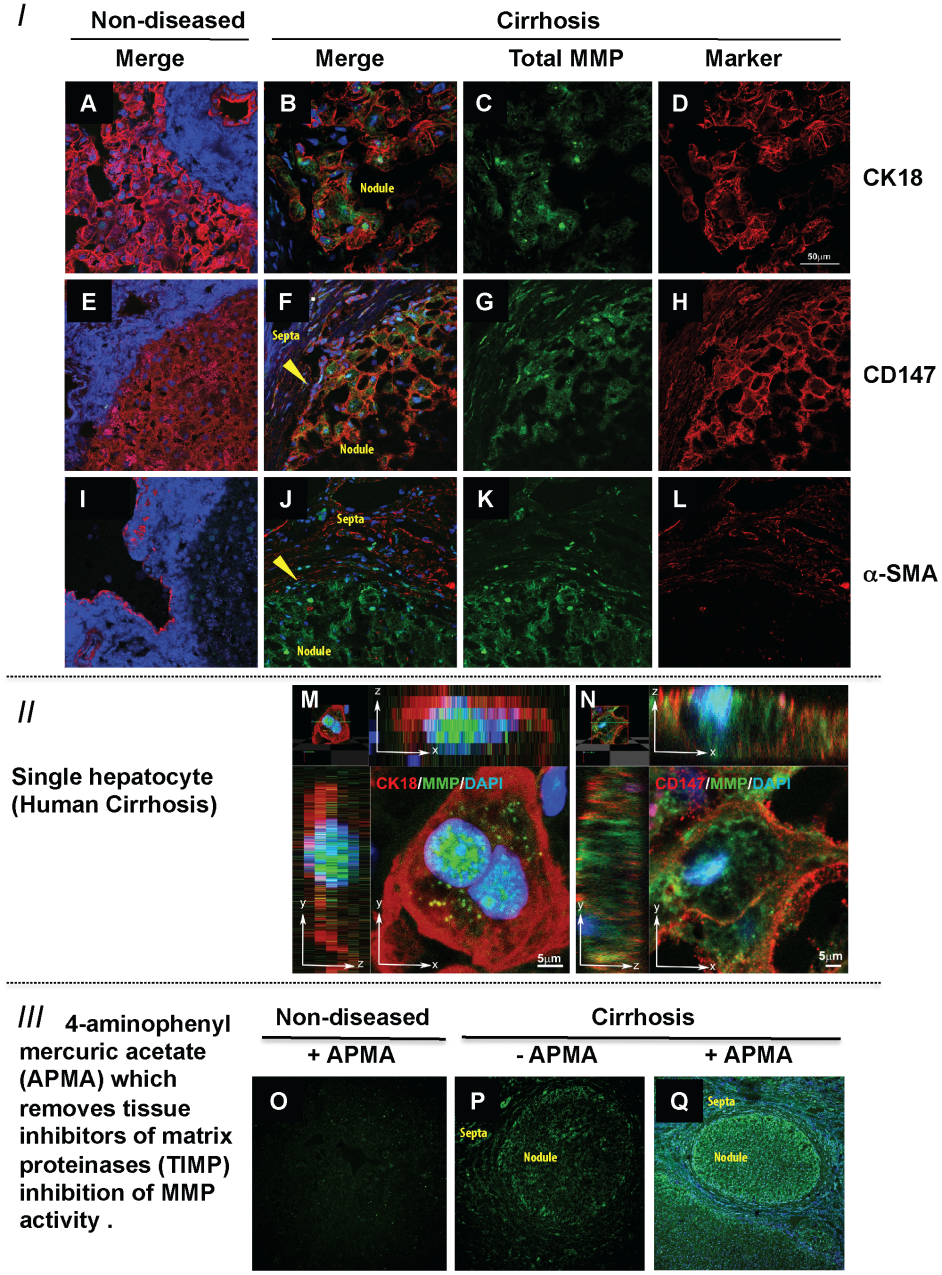
In-Situ Zymography of Matrix Metalloproteinase Activity and its Cellular Localisation in Liver Tissue. Co-localisation in human liver of MMP activity (green) with hepatocyte marker CK18 (red - Panels A, B, D and M) and CD147 (red - Panels E, F, H and N), nuclei are stained with Dapi (blue). MMP activity (green) can be seen within the cells (Panels B, F, M and N). Confocal microscopy shows an absence of MMP activity (green) in non-diseased tissue (Panels A, E and I) but abundant hepatocyte MMP activity in CK18 positive hepatocytes (Panels B and M), these binuclear cells also express CD147 (Panels F and N). Importantly, **α**-SMA (red) an activated HSC-marker is not expressed in non-diseased liver HSCs (Panel I) but is expressed in cirrhosis (Panels J and L). **α**-SMA (red) does not co-localise with MMP activity in green (Panel J). The effect of addition of aminophenylmercuric acetate (APMA) is shown in the third group of confocal panels (Panels O–Q). There is no TIMP regulated MMP activity in non-diseased liver (Panel O). In cirrhosis, basal MMP activity (Panel P) is increased markedly with APMA addition (Panel Q compared to P). In panels B, F, J, P and Q the hepatic nodule and fibrotic septa have been labelled. In addition the arrowheads in panels F and J highlight the boundary between the nodule and septa. Magnification for panels A-L is 63× and for panels is O-Q 10×.

*In-situ* zymography was used to co-localise MMP activity with markers of cellular origin in human liver sections. MMP activity (green) was readily observed across the lobules in cirrhotic tissue (Figure 2, Panels C, G, K) and was undetectable in non-diseased tissues (Figure 2, Panels A, E, I). Co-localisation studies, showed that MMP activity was found in CK18 positive hepatocytes (Figure 2, merged image Panel B) and CD147 (Figure 2, merged image Panel F) positive cells. Further, at higher magnifications using Z-stacks MMP activity clearly localised to nucleus and cytoplasm of both, CK18 positive (Figure 2, Panel M) and CD147 positive cells (Figure 2, Panel N). CK18 and CD147 positive MMP-expressing cells were binuclear and therefore easily identified as hepatocytes (Figure 2).

There was no significant MMP activity in non-diseased tissue (Figure 2 Panels A, E and I) and comparatively weak MMP activity seen in the fibrous septa compared to the hepatocyte in cirrhosis (Figure 2 Panels C, G, K and P). Importantly, abundant MMP activity in the septa of cirrhotic tissue was seen localised to the HSC but only after the addition of APMA, which removes the tissue inhibitor of matrix metalloproteinase (TIMP) blockade of MMP activity [35], [36] (Figure 2 Panel Q). The effect of addition of APMA to the non-diseased tissue is also shown (Figure 2, Panel O). The α-SMA positive cells including HSCs in the fibrous septa have diminished MMP activity in cirrhotic tissue compared to the hepatocytes within cirrhotic nodules (Figure 2 Panel P). However, the addition of APMA resulted in a marked increase in green fluorescence in the fibrous septa as well as the lobule (Figure 2 Panel Q compared to P). Therefore, as expected, MMPs are abundantly produced by HSC but their activities *in-vivo* are regulated by bound TIMPs [39], [40].

**Figure 3:**
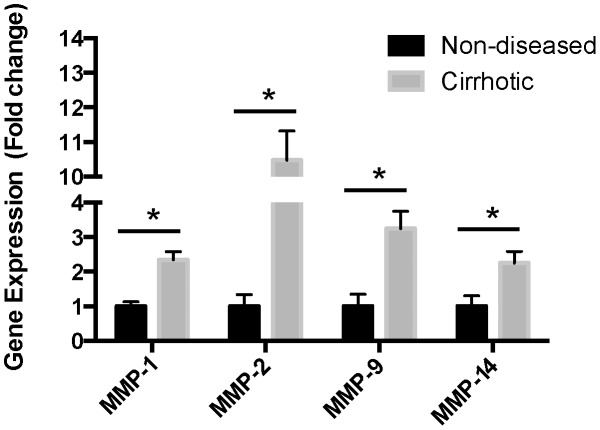
Expression of MMP mRNA in human liver. Quantitative PCR on whole liver tissue, non-diseased and HCV cirrhosis, of MMP-1, MMP-2, MMP-9 and MMP-14 mRNA (n = 4 per group). The expression of MMPs measured was significantly increased in cirrhosis compared to non-diseased controls, *p<0.05.

To confirm these results we performed qPCR on whole tissue and measured expression of selected MMPs (Figure 3). We found significant upregulation of MMP-1, MMP-2, MMP-9 and MMP-14 (Figure 3, p<0.05 and n = 4 per group).

### CD147 Expression in Human Cirrhotic Liver

**Figure 4:**
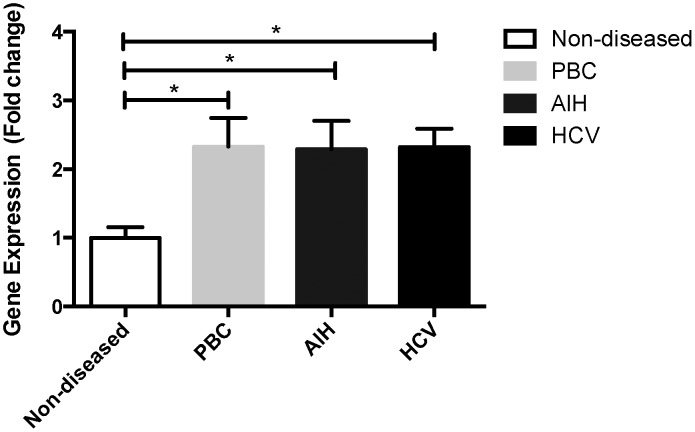
Expression of CD147 mRNA in human liver. Quantitative PCR on whole liver tissue, non-diseased as well as PBC, AIH and HCV cirrhosis of CD147 (splice variant 2) mRNA (n = 4 per group). CD147 was significantly increased in all cirrhotic specimens compared to non-diseased controls, *p<0.05.

**Figure 5:**
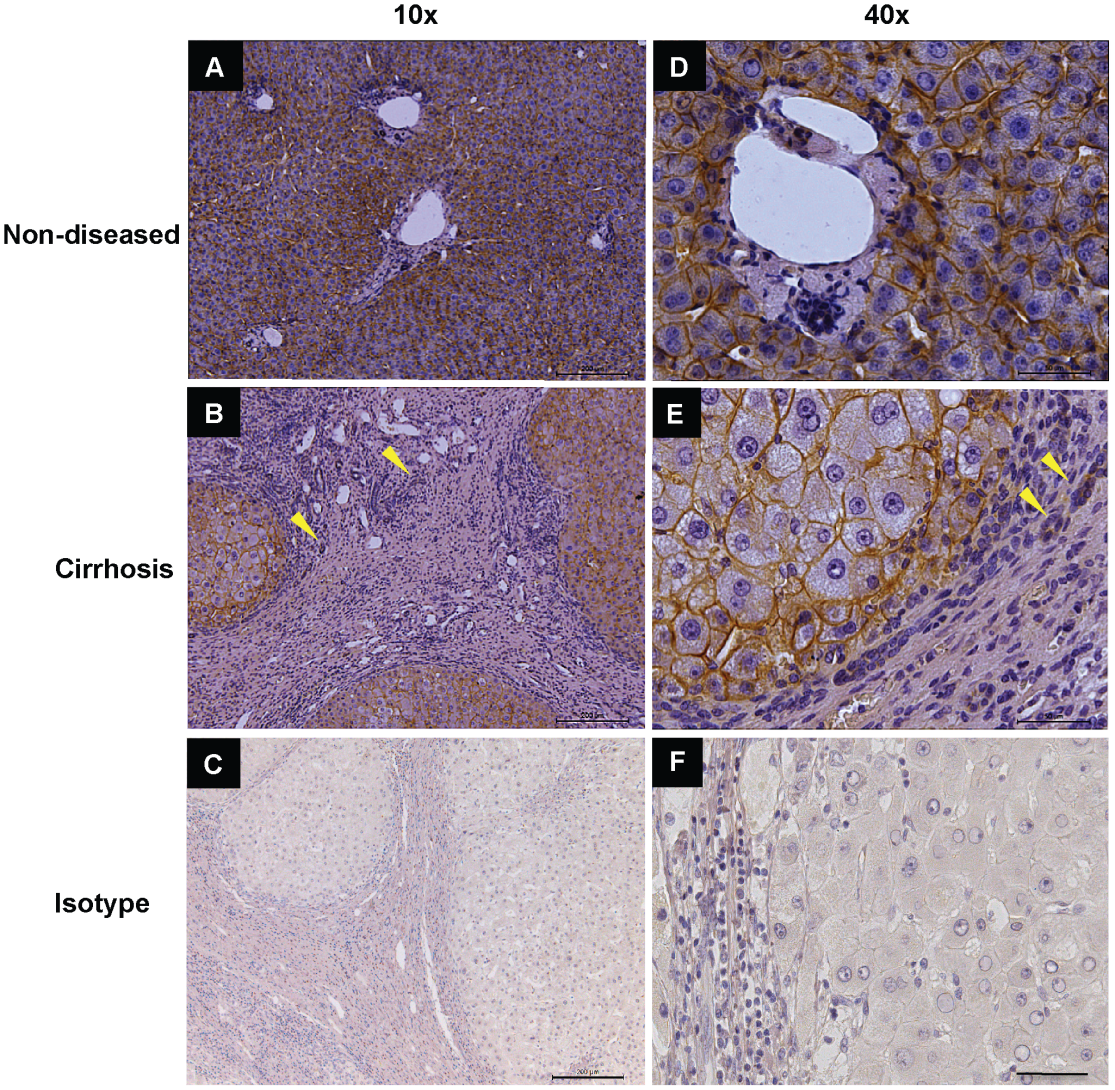
Immunohistochemistry of CD147 in Human Liver Tissue. The α-CD147 antibody (MEM-6/1) was used to stain non-diseased (Panels A and D) and cirrhotic ALD tissue (Panels B and E). CD147 is expressed by hepatocytes and bile ducts. No significant staining of HSC is seen in the fibrous septa, the only structures within the septa that are CD147 positive are bile ducts (see arrows Panel B and E). Isotype controls are shown in Panels C and F.

CD147 is a known regulator of MMPs, enzymes that play an important role in the remodelling of the ECM during liver injury and cirrhosis [3], [6], [41], [42]. We therefore wished to investigate whether MMPs within the injured liver would also be regulated by CD147. To quantitatively compare CD147 expression between non-diseased and cirrhotic liver tissues we used qPCR. A significant increase of greater than 2 fold in CD147 mRNA levels was seen in PBC, AIH and HCV explanted liver tissues compared to non-diseased liver tissue (Figure 4, p<0.05 and n = 4 per group). To further characterize CD147 localisation within the liver we stained non-diseased liver and end-stage cirrhotic liver with α-CD147 antibody. Figure 5 Panels A and D show CD147 expression in non-diseased tissue, which was equally distributed across the liver, while CD147 within the cirrhotic liver localised to cirrhotic nodules and not the fibrous bands (Figure 5 Panels B and E). No staining was observed in the isotype controls (Figure 5 Panels C and F). Figure 5 shows non-diseased donor and cirrhotic tissue from an ALD explant, we found identical staining for further non-diseased samples as well as PBC, PSC, HCV and AIH tissues (n = 3 per group, not shown). This staining pattern was also observed in murine models of liver injury (CCl_4_ and thioacetamide) using the commercially available and well characterized α-CD147 antibodies RL73.2 or G19 (data not shown). In non-diseased and cirrhotic liver, CD147 immuno-reactivity was consistent with cell membrane expression in hepatocytes. Importantly, no CD147 immuno-reactivity was observed in HSC within the fibrotic septa.

**Figure 6:**
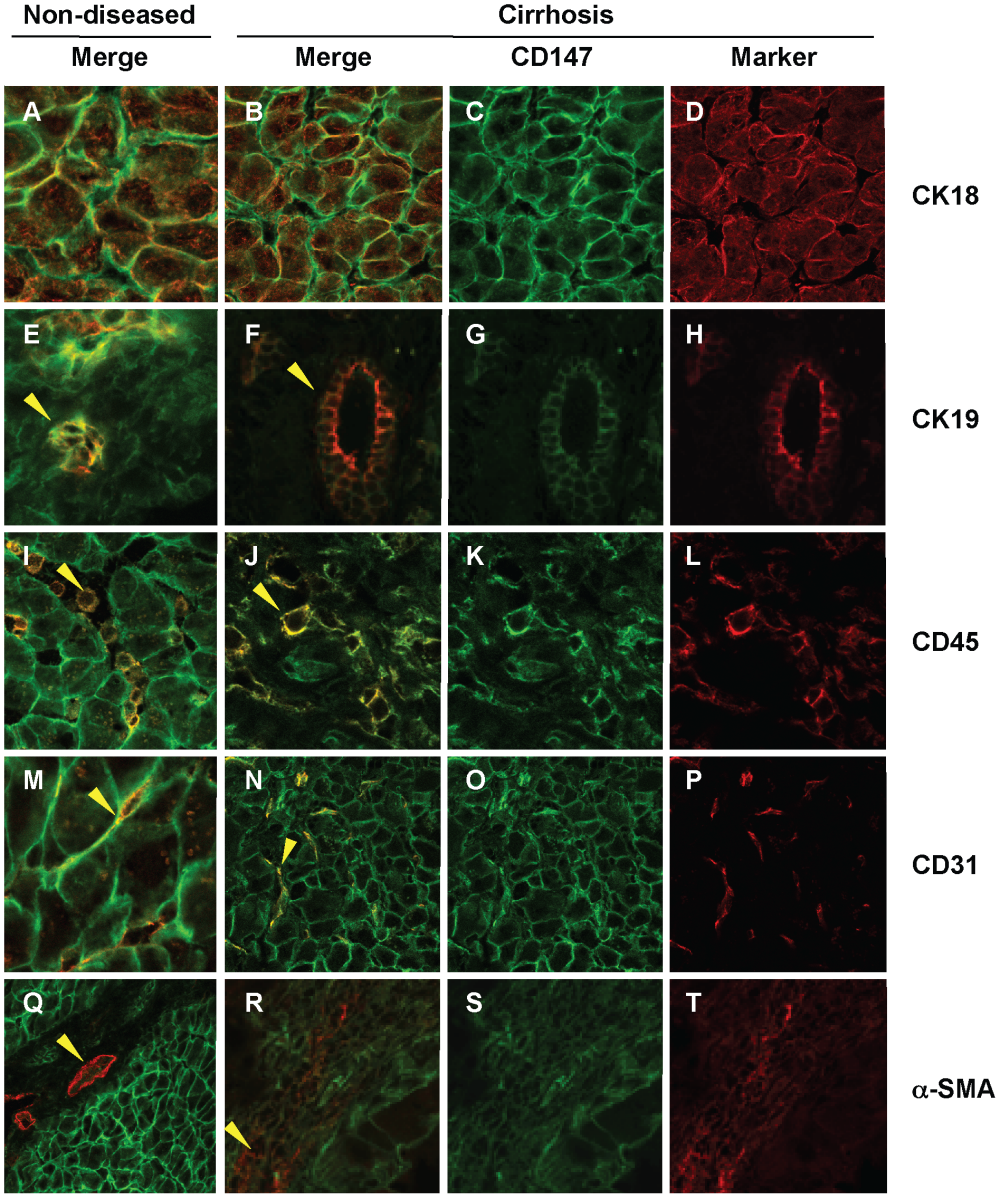
Co-localisation of CD147 and Liver Cell Markers in Human Liver Tissue. Liver sections were stained with liver cell makers (red) including CK18 (Panels A, B and D), CK19 (Panels E, F and H), CD45 (Panels I, J and L), CD31 (Panels M, N and P) and α-SMA (Panels Q, R and T) as well as CD147 (green, all except D, H, L, P and T). CK-19 positive bile ducts (Panels E, F and H) co-localise with CD147 (Arrowheads Panel E and F) in both non-diseased and cirrhotic tissue. Similarly, CD45 positive leukocytes (Panels I, J and L) co-localise with CD147 in both non-diseased and cirrhotic tissue (Arrowheads in Panel I and J). Further, CD31 endothelial cells (Panels M, N and P) co-localise with CD147 (Panel M and N) in both non-diseased and cirrhotic tissue. Finally, in the **α**-SMA positive series of images (Panels Q, R and T) vascular structures are seen stained as indicated by the arrowhead in Panel Q and HSC in fibrotic septa in cirrhosis (Arrowhead in Panel R). Importantly, no co-localisation of **α**-SMA and CD147 was seen in cirrhosis (Panel R). Merged images show co-localisation of CD147 with the liver cell markers (yellow). Magnification 63×.

To accurately determine which intrahepatic cell types express CD147, co-localisation of CD147 (green) and various liver cell markers (red) was examined in non-diseased liver and in explants of end-stage HCV cirrhosis (Figure 6). Hepatocytes (CK18^+^), cholangiocytes (CK19^+^), sinusoidal endothelial cells (CD31^+^) and leukocytes (CD45^+^) all showed CD147 immuno-reactivity at the cell membranes in cirrhotic (Figure 6 Panel B, F, J and N) and non-diseased tissue (Figure 6, Panels A, E, I and M). Importantly, CD147 did not co-localise with α-SMA, a marker of activated HSCs in the fibrous septa with chronic liver injury (Figure 6 Panel R). This pattern of staining was observed with different α-CD147 antibodies, the mAb MEM-6/1 in human tissue as shown here and also with RL73.2 and G19 in our murine liver injury models (data not shown). Further, in non-diseased tissue α-SMA staining was restricted to vascular structures and did not co-localise with CD147 (Figure 6 Panel Q).

### CD147 Regulation of MMPs in Human Hepatocytes

As the MMP mediator CD147 is expressed by hepatocytes we studied the functional importance of this gene in regulating hepatocyte MMP expression and activity. On immunoblot in both, human and mouse hepatocytes and liver tissue, CD147 is generally seen as 38 kDa (low glycoform, LG) and 54 kDa (high glycoform, HG) glycoforms, which are reduced to the 28 kDa non-glycosylated protein upon tunicamycin treatment of cells or PNGase F treatment of tissue lysates [43]. To investigate the role of CD147 in regulation of hepatocyte MMPs in humans we used the hepatocyte cell line pH 5CH8 [28], [29].

**Figure 7:**
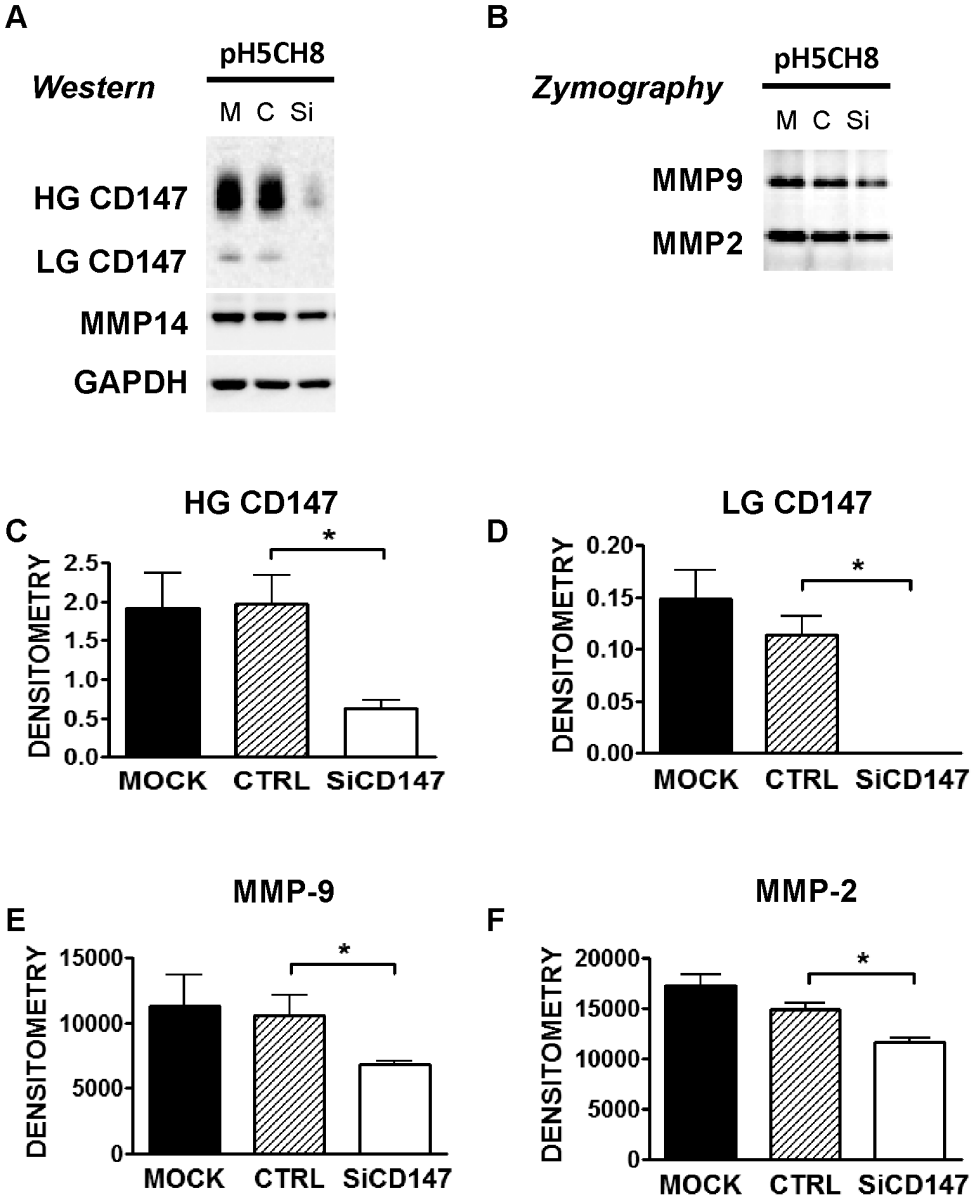
The Effect of Inhibition of CD147 on Matrix Metalloproteinase Expression and Activities in pH5CH8 Hepatocytes. Downregulation of MMPs with CD147 knockdown in a hepatocyte cell line *in-vitro*. pH5CH8 cells were transfected with siCtrl (C) or siCD147 (Si) oligonucleotides. Transfected cells and mock controls (M) were cultured in serum free conditions for 48 hrs. Shown in panel A are representative immunoblots of CD147 with the higher (HG CD147) and lower molecular weight (LG CD147) glycoforms, MMP-14 and GAPDH as loading control. Gelatin zymography of MMP-2 and -9 on the conditioned media from the same cells are shown in panel B. Densitometry was performed on the CD147 immunoblots and the results are shown as HG CD147 and LG CD147 forms normalised for GAPDH (n = 3, Panels C and D). Densitometric analysis of gelatin zymography of MMP-9 and MMP-2 are shown (Panels E and F). *p<0.05 using Mann-Whitney U t-test, compared to Mock (n = 3 for all groups).

Consistent with the observed minimal MMP activity seen in non-diseased liver by *in-situ* zymography, pH 5CH8 hepatocytes do not produce significant amounts of MMPs without stimulation. However, on exposure to the inflammatory mediator TNF these cells significantly upregulate MMP expression. This effect is analogous to the known *in-vivo* situation in advanced fibrosis [41], [44] and the situation documented in Figure 7. Compared with scrambled siRNA (C or CTRL) and untransfected control cells (M or mock) the siRNA targeting CD147 (siCD147) reduced HG-CD147 and LG-CD147 protein expression significantly (representative gel Figure 7, Panel A with quantitation in Panels C and D). This decreased protein expression was accompanied by a significant reduction in MMP-9 and MMP-2 activity (Figure 7, Panel B with quantitation in Panels E and F). Immunoblot analysis of MMP-14 in these cells also showed a decrease in protein level with CD147 knockdown, however this failed to reach statistical significance (graph not shown). These *in-vitro* results suggest a role for CD147 in regulation of hepatocyte MMP expression and activity.

### Matrix Metalloproteinases in Mouse Models of Liver Injury

**Figure 8:**
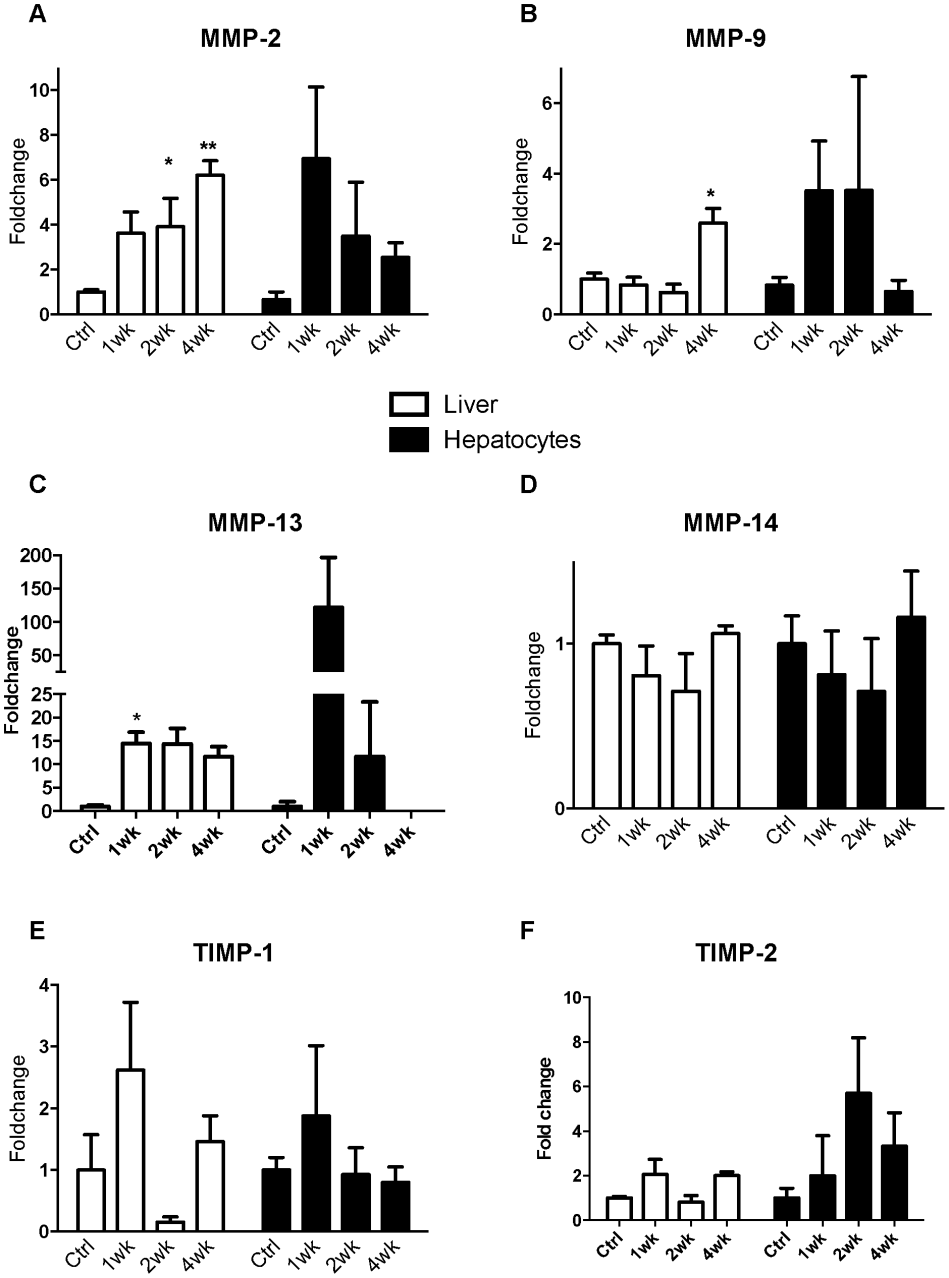
MMP and TIMP expression in whole liver and primary hepatocytes from CCl_4_ induced liver injury. Quantitative PCR of MMP and TIMP expression in whole liver and isolated primary hepatocytes. Cirrhosis was induced in a mouse model of liver injury (*C57bl/6*) with CCl_4_. RNA extracted from primary hepatocytes as well as whole liver were assessed at commencement of injury and weeks 1, 2 and 4. The expression of MMP-2 (Panel A), MMP-9 (Panel B), MMP-13 (Panel C), MMP-14 (Panel D), TIMP-1 (Panel E) and TIMP-2 (Panel F) was assessed by quantitative PCR (n = 4 per group, data expressed as mean and SEM. *p<0.05 and **p<0.01 relative to untreated).

The effect of CCl_4_ on development of liver fibrosis in mice and expression of MMPs and TIMPs was investigated (Figure 8). PSR staining is increased with time in the CCl_4_ model with bridging fibrosis and cirrhosis apparent at week 4 (not shown). In whole liver MMP mRNA expression is increased with severity of disease (Figure 8). More specifically, in the CCl_4_ model whole liver MMP-13 mRNA expression was significantly increased after 1 week of injury whereas the increase in MMP-2 and MMP-9 was delayed and MMP-14 was unchanged (Figure 8, Panels A-D). This pattern was similar to that seen at comparable stages in a thioacetamide model (data not shown). TIMP expression varied with injury in both whole liver and hepatocytes although there were no significant changes in expression (Figure 8, Panels E and F). From this data it is apparent that MMP expression is highly complex and is differentially regulated with progressive injury. Furthermore the observation that significant amounts of MMPs are expressed by hepatocytes with progressive liver injury supports a role for this cell in the ECM remodelling accompanying liver injury. Additionally, MMPs produced by hepatocytes are not as tightly regulated by TIMPs as are MMPs produced in the fibrous septa from HSC. We have already demonstrated this with APMA addition to the *in-situ* zymography (Figure 2, Panels P and Q). These results obtained from our mouse models are entirely consistent with our human data.

### The Role of CD147 in Liver Injury

**Figure 9:**
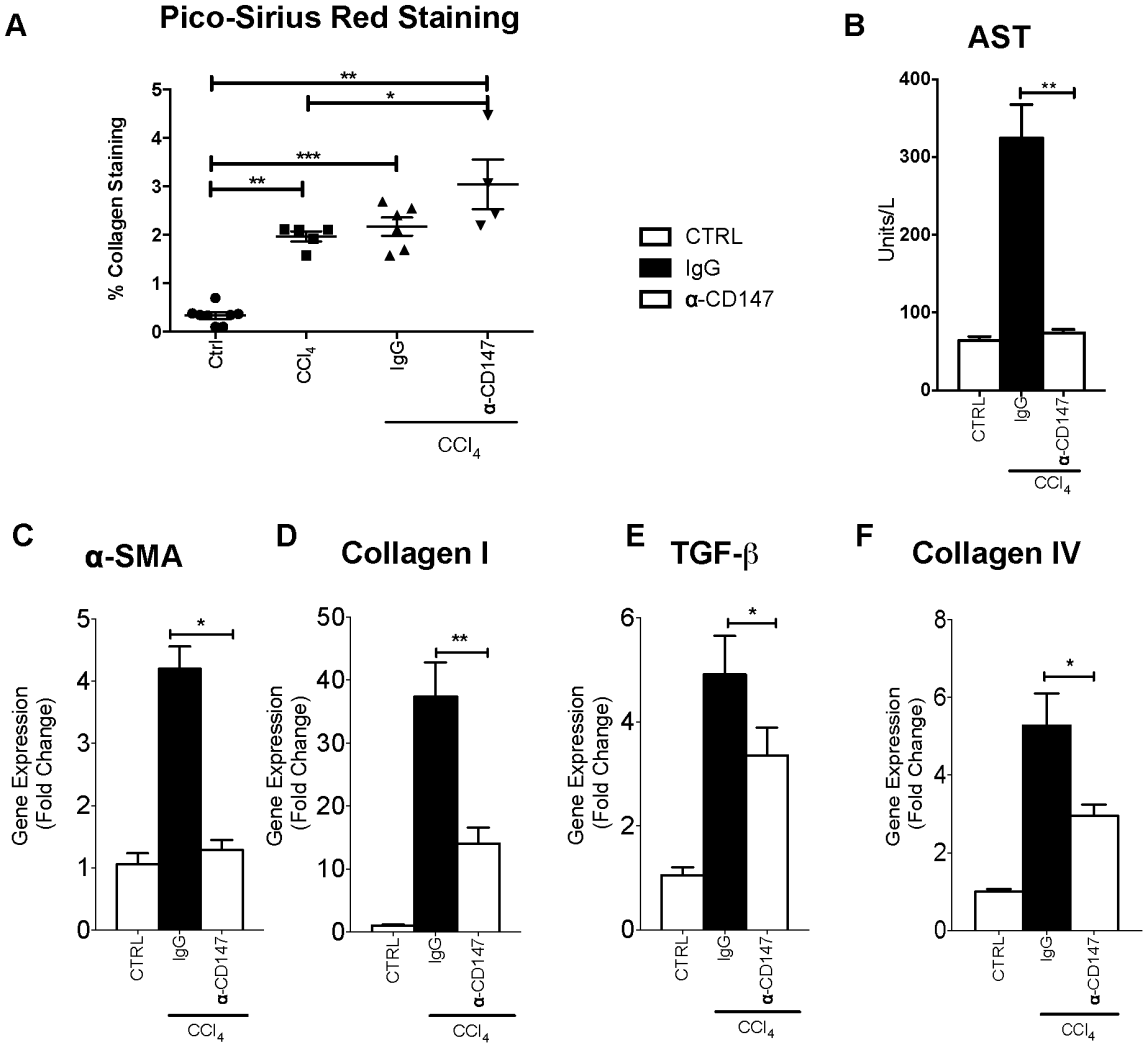
Injury in a mouse model of fibrosis with 4 weeks CCl_4_ and α-CD147 antibody intervention. Cirrhosis was induced in a mouse model of liver injury (*Balb/c*) with CCl_4_. An antibody targeting CD147 (RL73.2) was given twice weekly for 4 weeks, mice given an equal amount of IgG were used as control. There was no appreciable phenotype with either α-CD147 antibody or IgG control treatment in the absence of CCl_4_ (not shown). The injury groups are CCl_4_ (+/− isotype control) and CCl_4_ with antibody targeting CD147. Quantitative data of PSR staining is shown in panel A. Injury as assessed by AST (Panel B), α-SMA (Panel C), Collagen I (Panel D), TGF-β (Panel E) and Collagen IV (Panel F) were all significantly reduced in CCl_4_ injury with α-CD147 antibody intervention compared to the IgG control treated with CCl_4_. All groups are n = 4−6 with data expressed as mean and SEM. *p<0.05.

**Figure 10:**
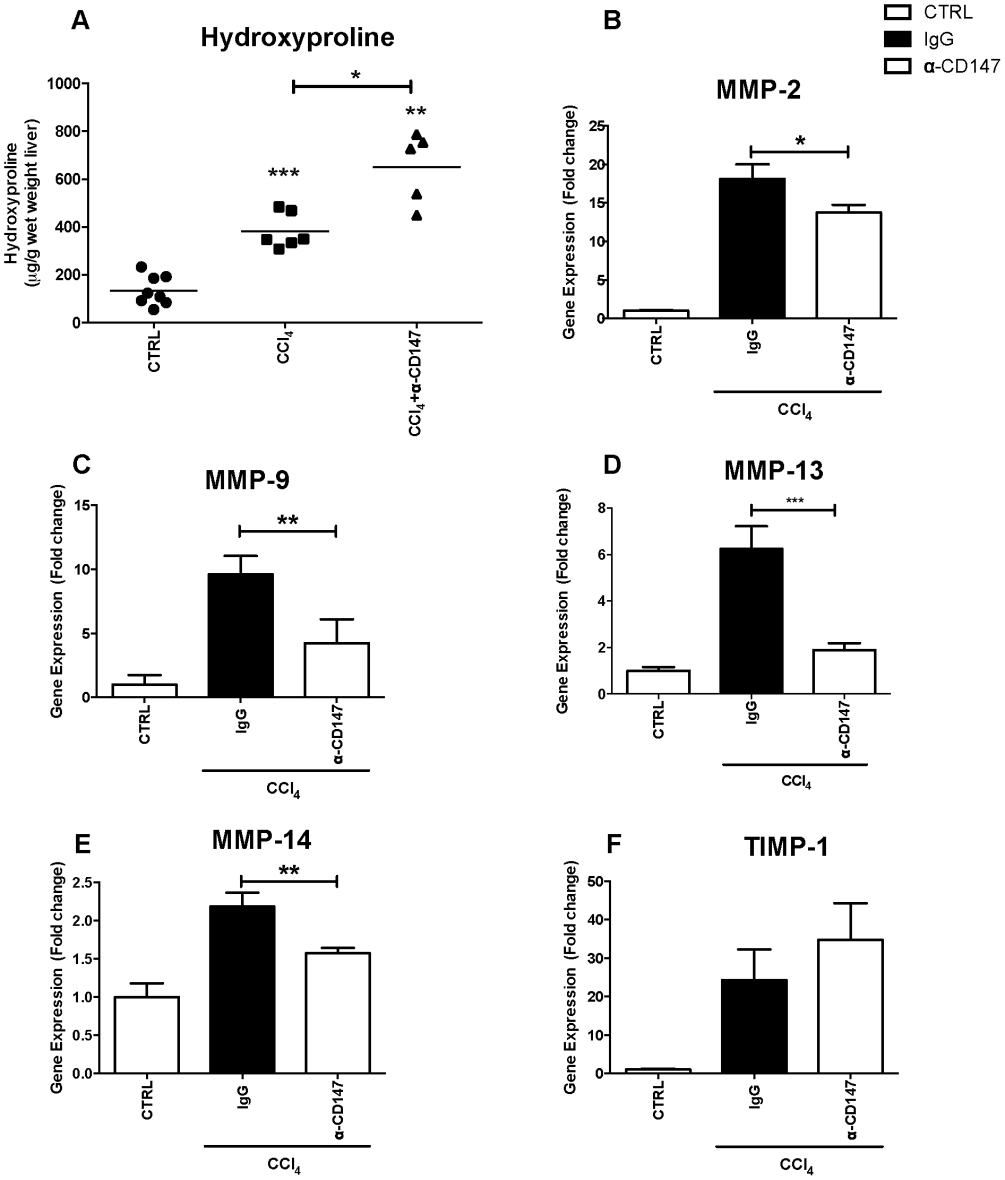
MMP expression in CCl_4_ induced liver injury with α-CD147 antibody intervention. Cirrhosis was induced in a mouse model of liver injury (*Balb/c*) with CCl_4_. An antibody targeting CD147 (RL73.2) was given twice weekly for 4 weeks, mice given an equal amount of IgG were used as control. There were no appreciable changes in MMP expression with either α-CD147 antibody or IgG control treatment in the absence of CCl_4_ (not shown). Tissue hydroxyproline was significantly increased with α-CD147 antibody treatment (Panel A). Compared with control MMP expression is significantly increased in the CCl_4_ injury with IgG group and reversed by intervention with α-CD147 antibody (Panels B–E). TIMP-1 expression was upregulated in injury but unaffected by intervention with α-CD147 antibody (Panel F) (n = 5−7 per group data expressed as mean and SEM. *p<0.05, **p<0.01 and ***p<0.001).

We wished to understand if CD147 is functionally important in regulating hepatocyte MMP expression and to determine if this has a functionally significant impact on intrahepatic fibrogenesis with liver injury (Figure 9 and 10).

To determine the contribution of CD147 in the ECM remodelling response to chronic liver injury *in-vivo*, the effects of an α-CD147 antibody were studied in the CCl_4_ model at 4 weeks. Treatment with CCl_4_ caused cirrhosis characterised by bridging fibrosis and was quantified by PSR staining (Figure 9, Panel A). Serum AST was increased by CCl_4_ compared to control (Figure 9, Panel B). Notably, as shown treatment with α-CD147 antibody ameliorated this increase and serum AST was significantly lower in the CCl_4_ and α-CD147 antibody group when compared to the CCl_4_ and IgG group (Figure 9, Panel B). The CCl_4_ injury alone (not shown) and CCl_4_ injury with IgG antibody were indistinguishable. To determine whether α-CD147 antibody could attenuate expression of mediators involved in fibrogenesis we examined the expression of **α**-SMA, collagen I and collagen IV and TGF- **β**, which were all increased with injury and significantly decreased by the α-CD147 antibody intervention (Figure 9, Panels C–F). The effect of α-CD147 antibody intervention on MMP expression and hydroxyproline concentration as a marker of collagen cross-linking was also studied (Figure 10). The antibody intervention inhibited the induction of MMP-2, MMP-9, MMP-13 and MMP-14 mRNA by CCl_4_ (Figure 10, Panels B–E). The reduced MMP expression with α-CD147 antibody intervention led to an increased accumulation of cross-linked collagen evident by significantly increased hydroxyproline concentration (Figure 10, Panel A) and PSR staining (Figure 9, Panel A). Together these results are consistent with MMP expression being regulated by CD147.

## Discussion

CD147 has been found to regulate MMPs in a variety of tissues, including human peripheral blood monocytes, human pulmonary fibroblasts and in rheumatoid arthritis [15], [21], [23]. Additionally, over decades CD147 has been associated with a diverse range of cancers such as melanoma, glioblastoma, breast or pancreatic cancer and recently cholangiocarcinoma as well as hepatocellular carcinoma (HCC), a liver cancer originating from hepatocytes [16], [17], [45]–[49]. The functions of CD147 are varied and depend on interacting proteins and cell types [50]–[58]. Many interacting proteins are matrix components or inflammatory mediators that are dramatically increased with injury (i.e. hyaluronan [50], intercellular adhesion molecule (ICAM)-1 [51]–[53], lymphocyte function-associated antigen (LFA)-1 [54]–[56] and CD43 [57], [58]).

Previous studies examining MMP expression in non-HSC populations, such as hepatocytes, have focused on regenerative responses and resolution of injury [41], [59]. Expression of MMPs by hepatocytes has been described previously associated with a regenerative response, and hepatocytes have been shown to increase expression of MMPs-2, -9 and -14 in association with injury resolution [12], [60]. Further, MMP-10 expression by hepatocytes as well cholangiocytes and macrophages is implicated in the ECM remodelling of progressive fibrosis [13]. Previous work and now the data in this manuscript demonstrate that hepatocytes with progressive fibrotic injury do produce a number of active MMPs capable of significant ECM remodelling.

In progressive fibrotic liver injury a number of mediators such as TNF are increased which can induce MMP expression [41], [44]. In primary hepatocytes, MMP-9 expression is increased with TNF and epidermal growth factor, both key molecular mediators increased in injury [41], [44] and involved in hepatocyte regeneration [59]. In our study, the *in-situ* zymography technique provided a powerful tool for confirming that hepatocytes produce MMPs that are active. Further, hepatocytes isolated from injured mouse livers expressed MMP-2, MMP-9, MMP-13 and MMP-14 mRNA at levels approximate to that seen in whole liver tissue. Together our results are consistent with hepatocytes being a major source of MMPs. Additionally, as expected, we also show that HSC in the fibrous septa are associated with active MMPs. Functionally this is likely to be important as hepatocytes, that are surrounded by abundant pericellular fibrosis, have reduced function [61]. In human cirrhotic liver samples *in-situ* zymography in the absence of APMA showed MMP enzyme activity across the nodule. Following the addition of APMA to remove TIMP inhibition, increased MMP activity was observed in the hepatocytes within nodules and the fibrous septa. These results in which APMA addition was used to determine the effect of TIMP inhibition on MMP activity are comparative but intriguing. They suggest that there is greater TIMP regulation of MMPs produced by HSC in the fibrous septa compared to those MMPs produced by hepatocytes. The significance of this result is unknown but we propose that this may represent on-going active ECM turnover by hepatocytes in the context of progressive injury. Further work will need to determine the functional importance of this observation.

The temporal evolution of MMP expression in whole liver and hepatocytes with injury is complex [8]. In both mouse models the most striking early gene up-regulation is seen in MMP-13 in whole liver. This is consistent with the change from normal to abnormal matrix turnover with injury as MMP-13 degrades fibrillar collagens especially collagen I. The early increase in MMP-13 we observed has been previously described and is implicated in the fibrogenesis associated with the initial phase of injury [62]. Importantly, this increase in MMP-13 is seen early and concurrent with significant **α**-SMA and collagen IV expression. Further, MMP-2 and MMP-9 were both found to be upregulated in primary hepatocytes after one week of CCl_4_ treatment. MMP-9 is primarily responsible for degradation of type IV and V collagens and is produced by HSC, inflammatory cells [42], [63] as well as being expressed in hepatocytes with injury as demonstrated in this study. In established end-stage injury with cirrhosis there is increased expression of MMP-2, MMP-9, MMP-14 as well as TIMP-1 and TIMP-2 [42], [64]. The differential expression of MMPs described in this study and by others [8] clearly implicates individual MMPs, the cell of origin, and the stage of injury in determining the final fibrotic phenotype. This impacts on the functional significance of target gene interventions in progressive fibrosis as this is dependent on the intrahepatic cell types expressing the gene, the stage of injury, and which MMPs are regulated by the target gene.

A key novel result from our work shows that MMPs produced by hepatocytes can be regulated by the membrane glycoprotein CD147. CD147 is a known mediator of both inflammation and ECM remodelling [18], [19], [23] and was found expressed abundantly on hepatocytes, with no significant staining evident through the fibrous septa on HSC. *In-vitro* studies of the pH 5CH8 hepatocyte cell line identified that MMP expression is partially regulated by CD147. The *in-vivo* data further supports a CD147-dependent role in mediating MMP dependent matrix remodelling. Following **α**-CD147 antibody intervention in the CCl_4_ injury model we have demonstrated a reduction in tissue injury characterised by reduction in **α**-SMA mRNA and necroinflammatory activity (AST). The reduction in inflammation may result from a reduction in ECM degradation products that can perpetuate HSC activation and the production of pro-inflammatory mediators such as TGF-**β** [2], [65]. Additionally, the reduction in necroinflammatory activity may reflect an additional anti-inflammatory effect of the **α**-CD147 antibody intervention given the near ubiquitous expression of this glycoprotein on leukocytes. The net effect with **α**-CD147 antibody intervention was a reduction in MMP expression but an increase in hydroxyproline, indicating greater collagen crosslinking. This is most likely due to the reduction in MMP-13 cleavage of predominantly type I collagen seen in abnormal ECM combined with the observed MMP-9 increase, which degrades predominantly normal type IV collagen. Further, MMP-14 is abundantly expressed by hepatocytes and studies of CD147 regulation of MMP-14 in tumour cells have found that a feedback loop exists, whereby MMP-14 cleaves CD147 from the cell surface to produce soluble CD147 ligand, which results in an autocrine regulation of the expression of both MMP-14 and CD147 [66], [67]. These observations support the hypothesis that in hepatocytes there is active production of MMPs and this is regulated by CD147 with progressive injury.

These studies have shown that hepatocyte-derived MMPs are capable of ECM turnover. Further, with reduced hepatocyte activity of MMPs there is accumulation of cross-linked ECM. Importantly, we have demonstrated that MMP expression can be regulated by the glycoprotein CD147. The novel finding of intrahepatic active hepatocyte MMP production, which is regulated by CD147, presents a new pathway that could be manipulated by possible future anti-fibrotic therapeutics.

## Supporting Information

Table S1 **Taqman probe sequences used for quantitative PCR.**
(DOCX)

Table S2 **Primer sequences used for quantitative PCR with Sybr Green.**
(DOCX)
