# Supplementary material for: Hepatocyte Produced Matrix Metalloproteinases Are Regulated by CD147 in Liver Fibrogenesis
Source: PLoS One. 2014 Jul 30;9(7):e90571. doi: 10.1371/journal.pone.0090571 (PMC4116334; doi:10.1371/journal.pone.0090571)
Supplement: Table S1 — Taqman probe sequences used for quantitative PCR. (DOCX) [file pone.0090571.s001.docx]

**Table S1**: Taqman Probes.

| Species | Gene | Applied Biosystems  Cat. No. | Product Size (bp) |
| --- | --- | --- | --- |
| Eukaryotic | 18S | Hs99999901_s1 | 187 |
| human | CD147 | Hs00942302_m1 | 223 |
|  | MMP-1 | Hs00233958_m1 | 133 |
|  | MMP-2 | Hs01548724_m1 | 96 |
|  | MMP-9 | Hs00957562_m1 | 67 |
|  | MMP-14 | Hs01037009_g1 | 92 |
| Mouse | β-actin | Mm01205647_g1 | 72 |
|  | α-SMA | Mm01546133_m1 | 88 |
|  | Basigin (CD147) | Mm01144228_g1 | 63 |
|  | MMP-2 | Mm00439498_m1 | 63 |
|  | MMP-9 | Mm00600163_m1 | 107 |
|  | MMP-14 | Mm01318966_m1 | 82 |
|  | MMP-13 | Mm01168713_m1 | 124 |
|  | TGF-β | Mm03024053_m1 | 108 |
|  | TNFa | Mm00443258_m1 | 81 |
