# Supplementary material for: Hepatocyte Produced Matrix Metalloproteinases Are Regulated by CD147 in Liver Fibrogenesis
Source: PLoS One. 2014 Jul 30;9(7):e90571. doi: 10.1371/journal.pone.0090571 (PMC4116334; doi:10.1371/journal.pone.0090571)
Supplement: Table S2 — Primer sequences used for quantitative PCR with Sybr Green. (DOCX) [file pone.0090571.s002.docx]

**Table S2:** Primer Sequences for Sybr Green Assay.

|  | Forward primer (5’-3’) | Reverse primer (5’-3’) |
| --- | --- | --- |
| mMMP-2 | AGATCTTCTTCTTCAAGGACCGG | GGCTGGTCAGTGGCTTGGGGTA |
| mMMP-9 | CCTTGGTGTAGCACAACAGC | ATACTGGATGCCGTCTATGTCG |
| mMMP-13 | AGCAGTTCCAAAGGCTACAACT | GGATGCTTAGGGTTGGGGTC |
| mMMP-14 | AGCACTGGGTGTTTGACGAA | CCGGTAGTACTTATTGCCCCG |
| mTIMP-1 | GGCATCCTCTTGTTGCTATCACTG | GTCATCTTGATCTCATAACGCTGG |
| mα-SMA | GCTGCTCCAGCTATGTGTGA | CCATTCCAACCATTACTCCCTGA |
| mTNF | ATGGCCTCCCTCTCATCAGT | CTTGGTGGTTTGCTACGACG |
| mTGFβ | GCTGAACCAAGGAGACGGAA | ATGTCATGGATGGTGCCCAG |
| m36B4 | TCGTTGGAGTGACATCGTCTT | TCTGCTCCCACAATGAAGCA |
